# Prion-like aspects of cerebral amyloidosis

**DOI:** 10.1186/1750-1326-8-S1-O21

**Published:** 2013-09-13

**Authors:** Mathias Jucker, Lan Ye, Sarah Fritschi

**Affiliations:** 1 Department of Cellular Neurology, Hertie Institute for Clinical Brain, Research, University of Tübingen, and German Center for Neurodegenerative Diseases (DZNE), D-72076 Tübingen, Germany

## 

The commonality of many neurodegenerative disorders is the predictable temporal occurrence and progression of specific aggregated proteins in the brain. The hallmark proteopathy is Alzheimer’s disease in which aggregated amyloid-β peptide (Aβ) is deposited in the brain parenchyma as amyloid plaques. Multiple evidence suggests that β-amyloidosis is induced by aggregated Aβ which can spread within and among brain regions and act as corruptive templates (seeds) that induce a chain-reaction of Aβ misfolding and aggregation. The insight that the prion paradigm may also apply to cerebral β-amyloidosis and other proteopathies suggests new avenues in search of biomarkers and novel therapeutic strategies.

